# A Cautionary Note on “A Cautionary Note on the Use of Ornstein Uhlenbeck Models in Macroevolutionary Studies”

**DOI:** 10.1093/sysbio/syad012

**Published:** 2023-05-25

**Authors:** Mark Grabowski, Jason Pienaar, Kjetil L Voje, Staffan Andersson, Jesualdo Fuentes-González, Bjørn T Kopperud, Daniel S Moen, Masahito Tsuboi, Josef Uyeda, Thomas F Hansen

**Affiliations:** Research Centre in Evolutionary Anthropology and Palaeoecology, Liverpool John Moores University, Liverpool, UK; Department of Biosciences, Centre for Ecological and Evolutionary Synthesis (CEES), University of Oslo, Oslo, Norway; Department of Biological Sciences and the Institutes of Environment, Florida International University Miami, Miami, FL, USA; Natural History Museum, University of Oslo, Oslo, Norway; Department of Biological and Environmental Sciences, University of Gothenburg, Göteborg, Sweden; Department of Biological Sciences and the Institutes of Environment, Florida International University Miami, Miami, FL, USA; GeoBio-Center LMU, Ludwig-Maximilians-Universität München, Richard-Wagner Straße 10, 80333 Munich, Germany; Department of Earth and Environmental Sciences, Paleontology & Geobiology, Ludwig-Maximilians-Universität München, Richard-Wagner Straße 10, 80333 Munich, Germany; Department of Integrative Biology, Oklahoma State University, Stillwater, OK 74078, USA; Department of Biology, Lund University, Lund, Sweden; Department of Biological Sciences, Virginia Tech, Blacksburg, VA, USA; Department of Biosciences, Centre for Ecological and Evolutionary Synthesis (CEES), University of Oslo, Oslo, Norway

## Abstract

Models based on the Ornstein–Uhlenbeck process have become standard for the comparative study of adaptation. Cooper et al. (2016) have cast doubt on this practice by claiming statistical problems with fitting Ornstein–Uhlenbeck models to comparative data. Specifically, they claim that statistical tests of Brownian motion may have too high Type I error rates and that such error rates are exacerbated by measurement error. In this note, we argue that these results have little relevance to the estimation of adaptation with Ornstein–Uhlenbeck models for three reasons. First, we point out that Cooper et al. (2016) did not consider the detection of distinct optima (e.g. for different environments), and therefore did not evaluate the standard test for adaptation. Second, we show that consideration of parameter estimates, and not just statistical significance, will usually lead to correct inferences about evolutionary dynamics. Third, we show that bias due to measurement error can be corrected for by standard methods. We conclude that Cooper et al. (2016) have not identified any statistical problems specific to Ornstein–Uhlenbeck models, and that their cautions against their use in comparative analyses are unfounded and misleading. [adaptation, Ornstein–Uhlenbeck model, phylogenetic comparative method.]

Based on many comments from editors and reviewers, we perceive a growing distrust of using Ornstein–Uhlenbeck models in macroevolutionary studies of adaptation. The Ornstein–Uhlenbeck process is the simplest possible stochastic model of trait evolution around optimal states, capturing a key ingredient at the core of evolutionary biology: the idea that species have different trait values due to adaptation to distinctive niches. We thus find this distrust surprising. Why are some evolutionary biologists giving these models the cold shoulder?

The bulk of the distrust seems to derive from Cooper et al. (2016), which is an increasingly cited (over 250 citations) source for why Ornstein–Uhlenbeck models should be avoided. Cooper et al. (2016) used simulations to claim two problems. First, by using statistical significance as a model-selection criterion, they claimed that Ornstein–Uhlenbeck models were chosen too frequently over Brownian motion on data generated by the latter process. Second, they claimed that this problem was amplified by measurement error. They then proposed “best practices” when fitting Ornstein–Uhlenbeck models to comparative data. Some of their recommendations are sound and general to any modeling of empirical data, such as critically interpreting model parameters instead of focusing on model fit, accounting for measurement error, and assessing the adequacy of the applied model. We disagree, however, with Cooper et al.’s (2016) claim to have detected specific problems with the Ornstein–Uhlenbeck method and we find their recommendations against the application of the method to be unfounded.

The impact of the Cooper et al. (2016) paper is unfortunate, not only because their analyses are irrelevant to the claims they make but also because their recommendations are likely to dissuade researchers from testing hypotheses of adaptation with models that are best cut out for this purpose. This sentiment is compounded by the peer review process, as indicated by the fact that all of us (the authors), and likely many others, have had Cooper et al.’s. (2016) recommendations incorrectly cited in reviews of papers or grant proposals as evidence for statistical problems with the use of Ornstein–Uhlenbeck models. Thus, we are once again (e.g., Martins 2000) in danger of replacing models designed for the problem at hand with general approaches that are harder to interpret and that often make assumptions that are inconsistent with the tested hypotheses (Hansen 2014; Uyeda et al. 2018; Moen et al. 2022). A formal rebuttal of Cooper et al. (2016) is therefore long overdue.

## The Ornstein–Uhlenbeck model in phylogenetic comparative studies

Hansen (1997) introduced the specific use of Ornstein–Uhlenbeck models to test hypotheses of trait adaptation to different niches mapped on a phylogeny. The Ornstein–Uhlenbeck process includes a deterministic movement of species trait values toward optimal states that can vary in accordance with environmental or ecological variables. Hansen (1997) termed these states “primary optima” and suggested interpreting them as the average of local optima (≈ trait means) for many species adapting to a given primary niche. The idea is that “secondary” selective factors (e.g., other, species-specific niche axes that also affect the trait) average out across species, leaving a common effect of the primary niche. Hypotheses about adaptation are tested by estimating the primary optima for different states of the environmental or ecological variables and asking if they differ as predicted by the hypotheses (see Hansen 2014 for a detailed explanation).

Mathematically, a simple Ornstein–Uhlenbeck process is described by the stochastic differential equation:


$$\begin{array}{l} dy=-\alpha (y-\theta )dt+\sigma dW, \end{array}$$ (1)

where *dy* is the change in a given species’ mean trait value, *y*, over a short time interval *dt*, *θ* is the primary optimum, *α* determines the rate of adaptation toward the primary optimum, *dW* represents independent normally distributed stochastic changes with mean zero and unit variance over a unit of time, and σ is the standard deviation of these latter changes. The σ-parameter can also be expressed in terms of the stationary variance of the process, $v=\sigma^{2}/2\alpha$, which can be interpreted as the variance among species within a niche after a long period of independent evolution. The method has been implemented to varying degrees in a number of software packages that allow fixed discrete or continuously evolving niches on the branches of a phylogeny (e.g., *OUCH*, Butler and King 2004; *SLOUCH*, Hansen et al. 2008; *MATTICE,*Hipp and Escudero 2010; *OUwie*, Beaulieu et al. 2012; *mvSLOUCH*, Bartoszek et al. 2012; *SURFACE*, Ingram and Mahler 2013; *phylolm*, Ho and Ané 2014a; *bayou*, Uyeda and Harmon 2014; *mvMORPH*, Clavel et al. 2015; *l1ou*, Khabbazian et al. 2016; *PhylogeneticEM*, Bastide et al. 2018; *PCMFit*, Mitov et al. 2020). This approach made it possible to investigate biological questions related to whether focal traits of taxa sharing a given niche tend to be more similar compared to those of taxa sharing a different niche, as well as to estimate the level of adaptation and phylogenetic inertia in the clade (see Hansen 2014; Mahler and Ingram 2014; O’Meara and Beaulieu 2014; Moen et al. 2022 for general reviews).

While Cooper et al. (2016) stressed the α-parameter, we focus our discussion on a transformation of this parameter, the phylogenetic half-life. Calculated as $t_{1/2}=\ln\left(2\right)/\alpha$, the half-life has units of time and a more transparent biological meaning. The half-life can be interpreted as the average time for a trait to evolve halfway from an ancestral state toward a new optimum. Thus, it indicates how long it will take before adaptation to a new regime is more influential than constraints from the ancestral state. Given a phylogeny scaled to unit height, a half-life of unity would mean that a species starting to evolve in a new niche at the root of the phylogeny would be expected to have traversed half the original distance to the new primary optimum when it has reached the tip of the phylogeny. This can usually be interpreted as strong phylogenetic inertia and slow adaptation. A shorter half-life would mean faster adaptation and a half-life of zero would mean instantaneous adaptation and thus no phylogenetic correlation in species’ trait residuals. Half-lives in excess of phylogeny height mean that the process is increasingly resembling Brownian motion, as *α*—and thus the deterministic component of Equation (1)—asymptotes toward zero. A pure Brownian motion has a half-life of infinity.

## A cautionary note on model relevance

Throughout their article, Cooper et al. (2016) analyzed single-optimum Ornstein–Uhlenbeck processes to reach their conclusions regarding the use of the models in comparative studies in general. Single-optimum models are just that—they estimate a single constant optimum across the phylogeny. Thus, they assume that all species are adapting toward the same primary optimum.

However, *the* main utility of Ornstein–Uhlenbeck models in comparative analyses is to test adaptive hypotheses by fitting two or more regime-specific optima (Hansen 1997; Butler and King 2004; Beaulieu et al. 2012). Even the simple example in Hansen (1997) that introduced the method estimated distinct optima for grazers and browsers to assess the role of diet on hypsodonty in horses. As Cooper et al. (2016) did not consider multiple optima, it is unclear how their results pertain to the main application of the method.

The restriction to single-optimum models limits the generality of the results presented in Cooper et al. (2016). First, the estimation accuracy of the α parameter may differ between models with single and multiple optima. Shifts among different optima lead to more deterministic dynamics and thus more information about the α parameter. This is particularly true when optima are well separated and have several origins (Beaulieu et al. 2012; Boettiger et al. 2012; Ho and Ané 2013, 2014b; Cressler et al. 2015; Bartoszek et al. 2022). Second, inferences about adaptation are not based on the α-parameter, but on the primary optima, which are estimated as fixed effects in an ANOVA or regression, and their estimation accuracy can be substantial even with as few as ten species (Cressler et al. 2015). For example, Moen et al. (2022) discuss the advantages of multiple-optimum models and illustrate their utility, even with few species, using data on thermal physiology from just 12 species of tree frogs. By comparing different hypotheses, they identified an Ornstein–Uhlenbeck model with two primary optima for critical thermal minimum as the best explanation for how tropical tree frog species were able to colonize temperate zones. For multivariate traits, which require more data, Bartoszek et al. (2022) provide compelling examples of how rather complex hypotheses about coadaptation and biological trade-offs can be distinguished with multiple-optimum models fitted to data from 100 species or less.

Single-optimum models have two uses in evolutionary biology. First, they are used to describe patterns of evolution in evolutionary time series (e.g., Hunt et al. 2008; Lo Cascio Sætre et al. 2017; Voje 2020), which include information on the ancestral state and other traits that are usually unavailable in analyses based on comparative data from extant species. Second, they are used to model phylogenetic signal in residual variation in comparative studies not involving adaptation (e.g., allometry; Grabowski et al. 2016). In such analyses, it is not uncommon to use relative model fit to distinguish between trait dynamics described by a single-optimum Ornstein–Uhlenbeck model and a Brownian-motion model. Although Cooper et al.’s. (2016) results have some relevance to this practice, investigating performance not only on data generated by a Brownian motion but also from Ornstein–Uhlenbeck processes with a range of α-values—as has been done in previous studies of the method’s performance (e.g., Beaulieu et al. 2012; Boettiger et al. 2012; Ho and Ané 2013, 2014b; Cressler et al. 2015; Cornuault 2022)—would have provided a more relevant contrast.

The phylogenetic half-life is the canonical measure of phylogenetic signal in the Ornstein–Uhlenbeck framework and can be informative about the evolutionary processes that generated the data. We emphasize, however, that phylogenetic signal estimated from single-optimum models should not be used to select the evolutionary model for a comparative analysis of adaptation. A strong, Brownian-motion-like phylogenetic signal frequently occurs in data when related species adapt toward similar niches (Labra et al. 2009; Hansen 2014). Yet, such a pattern does not necessarily indicate a similar phylogenetic pattern in the residual deviations of species from the estimated optima. It is essential to understand that only phylogenetic correlations in model residuals should be accounted for in comparative inference (Hansen and Orzack 2005; Labra et al. 2009; Revell 2010; Hansen 2014). The α-parameters reported in Table 2 of Cooper et al. (2016) are estimates of phylogenetic signal in their simulated species data and thus do not address the idea of testing for systematic differences in primary optima for species adapting to different niches.

## Revisiting the simulations and parameter interpretations of single-optimum models

To produce their main results, Cooper et al. (2016) first generated phylogenies of varying size (25–1000 tips) and simulated the evolution of a single trait on each phylogeny under a simple, constant-rate Brownian-motion model of evolution. They then fitted a single-optimum Ornstein–Uhlenbeck model and a Brownian-motion model to the resulting data and compared them with a likelihood-ratio test. They recorded the proportion of times the likelihood-ratio test rejected Brownian motion in favor of the Ornstein–Uhlenbeck model. Cooper et al. (2016) argued that this proportion, which they termed the “rejection rate” in their Table 2, was “unacceptably high” in some circumstances. In consequence, they recommended that Ornstein–Uhlenbeck models should not be applied with fewer than 200 tips. They also recommended considering Bayesian approaches. While considering Bayesian approaches is reasonable (see Uyeda and Harmon 2014; Ross et al. 2016; Uyeda et al. 2017; Cornuault 2022), they are not alternatives to models like the Ornstein–Uhlenbeck process. Instead, they are simply statistical approaches to fitting the models. In any case, the key conclusions that Cooper et al. (2016) used to recommend caution against fitting Ornstein–Uhlenbeck models are not supported by their results. There are two main reasons for this.

First, by only simulating Brownian motion and only considering Type I error rates, Cooper et al. (2016) neglected to interpret the estimated parameters. Many cases of Type I error will be associated with best estimates of  α corresponding to relatively long half-lives, and in this situation, the dynamics predicted from the fitted Ornstein–Uhlenbeck model are similar to Brownian motions. Indeed, making a sharp distinction between Ornstein–Uhlenbeck and Brownian-motion processes unhelpfully discretizes a continuum. Finding instances of Type I error, in which the Ornstein–Uhlenbeck model is preferred over the Brownian-motion model, is not an argument against using Ornstein–Uhlenbeck models. Rather, it supports one of the general recommendations Cooper et al. (2016) made but did not heed: a comparative analysis should include careful interpretation of parameters. In fact, Cooper et al. (2016) observe that “scrutiny of the model parameters suggest that the favored Ornstein–Uhlenbeck model is biologically indistinguishable from Brownian motion” (p. 67) but fail to see that this undermines their own criticism of the method. In other words, a researcher who makes inferences not exclusively based on significance or model-selection criteria, but also considers the meaning of the estimated parameters would still conclude that evolution is similar to Brownian motion when confronted with parameter estimates showing an Ornstein–Uhlenbeck model with a long half-life.

This point is underscored by the simulations presented by Cressler et al. (2015), who showed the inverse result of Cooper et al. (2016), namely that Brownian motion could be chosen preferentially over Ornstein–Uhlenbeck models when data were generated from the latter with long phylogenetic half-lives. Unsurprisingly, “errors” are common when choosing between models that are essentially equivalent. This is especially true when the choice is conducted at the boundary of parameter space under the likelihood-ratio test, as in Cooper et al. (2016). When the null model lies on the boundary of the parameter space of the alternative model, the likelihood-ratio test is less reliable because the regular distributional properties of its statistic (see below) no longer hold (Self and Liang 1987; see also Ota et al. 2000; Dominicus et al. 2006). This is not an issue with the Ornstein–Uhlenbeck process but with the inferential setup adopted by Cooper et al. (2016), and it would apply similarly to any of the alternative models used to measure phylogenetic effects (e.g. Lynch 1991; Housworth et al. 2004).

Even in situations with long half-lives, there are benefits to estimating parameters with the more general Ornstein–Uhlenbeck models. In these situations, estimates of optima become inaccurate, but the model can be reparametrized to estimate regime-specific evolutionary trends (Hansen 1997). In fact, a multi-optimum Ornstein–Uhlenbeck process does not converge to simple Brownian motion when the half-life becomes long. Instead, it converges to Brownian motion with trends influenced by the regimes. These trends can be accurately estimated even when the conventional α and θ parameters are individually uncertain (Hansen 1997; and see Grabowski et al. 2023 for a recent example).

Second, Cooper et al. (2016) do not, in our opinion, fully appreciate that the parameters in these models have units. The unit of α, for example, is the inverse of time, which can be characterized in years, generations, or height of the phylogeny. Cooper et al. (2016) simulated trees conditional on the number of taxa, which produces trees of different heights. Though they sometimes imply in their article that they rescaled trees to unit heights, their published code does not do so. This means that the variation in their estimated α values incorporates variation in tree height across their simulated datasets. In other words, all the tables from Cooper et al. (2016) reporting the median and 95% quantiles for α across their simulations (e.g., their Table 2), which appear to show substantial variation in α per run, are inflated by variation in the height of the phylogeny, compromising their interpretation.

Using the available R code from Cooper et al. (2016), we recreated one of the main results of their study to investigate how these issues may have influenced their interpretations. This analysis explored whether the number of taxa in the phylogeny across varying extinction rates affects the relative fit of Brownian motion versus the Ornstein–Uhlenbeck model. We first reconstructed birth–death trees with varying numbers of tips (25, 50, 100, 200, 500, 1000) and relative extinction rates (0%, 25%, 50%, 75%). We simulated these trees with the function *tess.sim.taxa* from the R package TESS (Höhna et al. 2016). This function is the updated version of the now defunct *sim.globalBiDe.taxa* that Cooper et al. (2016) used. The relative extinction rate determines how branching events are distributed through time. Cooper et al. (2016) called these “d/b” ratios for death/birth, and we followed that style here but note that this is also termed the relative extinction rate, or ϵ (Magallón and Sanderson 2001). An ϵ=0 (the Yule model) results in an exponentially increasing number of branching events through time. By increasing the relative extinction rate, the branching events are skewed more toward the recent part of the phylogeny (“tippy” trees). We scaled all simulated trees to unit height so that phylogenetic half-lives from our simulations were comparable and interpretable as proportional to the total tree height. We then simulated data following Brownian motion with a constant rate parameter of *σ*^2^ = 1, using the *sim.char* function from the *geiger* package. All simulations were replicated 50 times, producing a total of 1200 simulation replicates (i.e. 6 tree sizes × 4 extinction rates × 50 replicates).

We first estimated parameters following the approach of Cooper et al. (2016) with the *transformPhylo.ML* function from *motmot* (Thomas and Freckleton 2012). We next used the same likelihood-ratio test as Cooper et al. (2016) to determine which model was better supported. We did this to produce results more directly comparable to theirs, although as noted above, we recognize that a proper likelihood-ratio test, in this case, would follow a procedure more similar to that outlined by Ota et al. (2000). We used a likelihood ratio (3.84) that corresponds to the critical value at 5% significance level under a $\chi^{2}$ test with one degree of freedom. We also used the same simulated data to estimate half-lives using the function *slouch.fit* from *SLOUCH* (Hansen et al. 2008; Kopperud et al. 2020), which allows one to estimate and visualize uncertainty in parameter estimates. The R code used to run our simulations is available at: https://github.com/mark-grabowski/C-Reply.

Our results are shown in Table 1. The rejection rate appears to show some of the same issues espoused by Cooper et al. (2016). Increasing the number of tips and decreasing the relative extinction rate generally leads to a lower rejection rate of Brownian motion. Mirroring Table 2 in Cooper et al. (2016), our Table 1 includes median and 95% quantiles, but is presented as phylogenetic half-lives, rather than as *α*-values. The median half-life for all simulations across all trees is well above phylogeny height, indicating that Brownian-motion-like dynamics dominate these results, but the lower 95% quantile includes relatively short phylogenetic half-life values for the smallest trees, indicating that fast Ornstein–Uhlenbeck dynamics were preferred in some cases. The highest rejection rate in our simulations was for a tree with 25 tips and an extinction rate of 75%, with a rejection rate of 16% (i.e., Brownian motion was rejected in eight of fifty simulation replicates).

**Table 1.** Rates of misinferring Ornstein–Uhlenbeck dynamics from data generated under Brownian motion across a range of tree sizes (number of tips) and birth–death models with varying relative extinction rates (*d*/*b*), as in Cooper et al. (2016)^a^

*d/b*	Tree size	Rejection rate	Median *t*_1/2_ [95% quantiles]	Qualitative error rate	Uncertain error rate
0	25	12%	1.66 [0.13–∞]	4%	2%
0	50	4%	1.60 [0.44–∞]	0%	2%
0	100	4%	2.85 [0.60–∞]	0%	0%
0	200	2%	5.74 [0.87–∞]	0%	0%
0	500	8%	9.92 [1.18–∞]	0%	0%
0	1000	6%	∞ [1.40–∞]	0%	0%
0.25	25	12%	1.40 [0.21–∞]	2%	4%
0.25	50	8%	2.13 [0.23–∞]	4%	0%
0.25	100	4%	1.85 [0.46–∞]	2%	0%
0.25	200	4%	3.79 [0.82–∞]	0%	2%
0.25	500	10%	8.94 [0.99–∞]	0%	2%
0.25	1000	2%	24.89 [1.51–∞]	0%	0%
0.5	25	8%	1.85 [0.22–∞]	2%	4%
0.5	50	10%	1.91 [0.22–∞]	4%	4%
0.5	100	2%	2.33 [0.57–∞]	0%	0%
0.5	200	4%	3.65 [0.69–∞]	0%	2%
0.5	500	4%	4.95 [1.23–∞]	0%	0%
0.5	1000	2%	12.39 [1.99–∞]	0%	0%
0.75	25	16%	1.33 [0.14–∞]	2%	10%
0.75	50	14%	1.92 [0.29–∞]	2%	6%
0.75	100	6%	2.72 [0.54–∞]	0%	0%
0.75	200	6%	5.13 [0.72–∞]	0%	0%
0.75	500	8%	72.86 [1.29–∞]	0%	0%
0.75	1000	4%	12.40 [1.73–∞]	0%	0%

^a^The column labeled “Rejection rate” gives the frequency at which Brownian motion is rejected according to the significance test used by Cooper et al. (2016). In addition, we report the median and 95% quantiles (across simulation replicates) of the estimated half-lives in units of tree height. The two last columns indicate rates of misinference when considering parameter estimates with uncertainty. The column labeled “Qualitative error rate” gives the percent of cases when both the best estimate and the upper limit of the 2-unit support interval of the half-life are less than one. These would be qualitative errors of inference. The column labeled “Uncertain error rate” gives the percent of cases when the best estimate of half-life is less than one, but the upper limit of the support interval is between one and three. These would be cases where a wrong model is found but reported to be uncertain. Note that both the qualitative and the uncertain error rates are much lower than the significance-based error rate. All rates are based on 50 simulation replicates per row.

On the surface, these results seem worrying, but the picture changes when we examine parameter estimates and uncertainty in the individual simulation replicates (Table S1). In the eight cases discussed above in which the Ornstein–Uhlenbeck model was preferred using *motmot*, the maximum-likelihood estimate of the half-life was usually far below tree height. Looking at these same runs using *SLOUCH*, however, shows that in seven of the eight cases, the 2-unit support interval extended above unity. In other words, these results correctly tell us that there is limited evidence to support an Ornstein–Uhlenbeck process that is qualitatively different from Brownian motion.

To reinforce this point, we added two new statistics to the rejection rate of Cooper et al. (2016). The “Qualitative error rate” shows the frequency at which both the best estimate and the upper limit of the 2-unit support interval for the half-life are less than unity. This simulates a situation in which a researcher considering the meaning and uncertainty of parameter estimates would reach a qualitatively wrong conclusion. The “Uncertain error rate” shows the frequency at which the best estimate of the half-life is less than unity, but the upper limit of the support interval is between one and three. This simulates a situation in which the researcher would not only reject Brownian motion and support meaningfully different Ornstein–Uhlenbeck models but also report that the evidence for this conclusion is modest. Our results in Table 1 show that both rates are always far below the significance-based rejection rate of Cooper et al. (2016), and quickly go to zero as the number of species increases.

**Figure 1.**
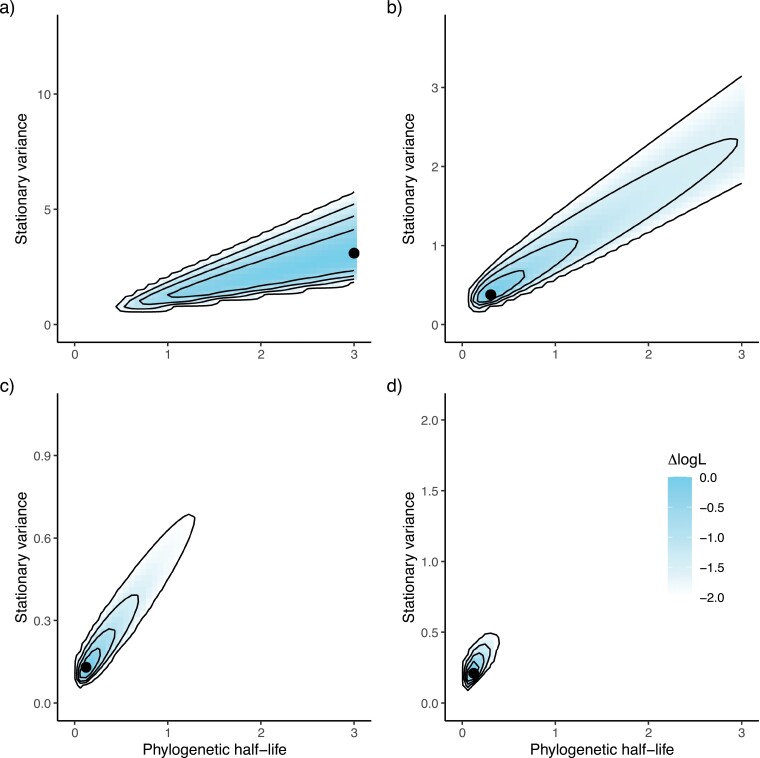
Support (log-likelihood) surfaces for phylogenetic half-lives (*t*_*1*/*2*_ = ln(2)/α) and stationary variances ($v=\sigma^{2}/2\alpha$) for four replicates. The trait data were simulated under a Brownian-motion model and fitted as an Ornstein–Uhlenbeck process on 25-tip trees simulated under a birth–death model (*d*/*b* ratio = 0.75). All trees were scaled to unit height. In each figure, the black dot is the maximum-likelihood estimate. The distance between the dot and the surrounding line, and the lines relative to each other, is 0.5 log-likelihood units. Run (a) depicts support for the Brownian-motion model. Runs (b)–(d) show increasing support for the Ornstein–Uhlenbeck model. A half-life above one indicates that the process is approaching Brownian motion and a half-life of three means that the model is dynamically similar to Brownian motion. The non-shaded parts of the figure correspond to estimates that were more than two support units worse than the best estimate. The cases a, b, c, and d correspond to simulation replicates 151, 164, 194, and 189, respectively, in Table S1.

Figure 1 shows the log-likelihood support surfaces from four individual runs of this “worst-case” simulation. Figure 1a shows the pattern seen in most runs—the maximum-likelihood estimate for the half-life is around or substantially greater than tree height, and thus the best model indicates a pattern similar to Brownian motion. The other three panels show patterns with varying degrees of support for an Ornstein–Uhlenbeck model. Figure 1b, c have maximum-likelihood half-life values of 31% and 12% of tree height, which indicate a model qualitatively different from Brownian motion. The former (Fig. 1b) has a ridge of support running out toward infinity, however, indicating that the evidence against Brownian motion is not strong. In the latter (Fig. 1c), the 2-unit support interval extends out to 129% of tree height, making it a case of the “Uncertain error rate.” Here, a wrong inference of Ornstein–Uhlenbeck dynamics would be made, but the possibility of relatively slow dynamics resembling Brownian motion is not definitely rejected. Figure 1d shows a case of the “Qualitative error rate” in which strong evidence for moderately fast Ornstein–Uhlenbeck dynamics would be reported. This particular run is responsible for the 2% qualitative error rate result for this simulation (i.e., it occurred once in 50 replicates).

None of these results deny that estimates of phylogenetic half-lives, and phylogenetic signal more generally, are often inaccurate for small phylogenies. Reporting uncertainty with support intervals, or Bayesian posterior distributions, is instrumental. In cases with high uncertainty, Hansen (1997; Hansen et al. 2008) recommended a qualitative approach in which inferences about adaptation, or other main effects, are reported conditionally on fixed values of half-lives. For example, a strong inference could be made if a result were to hold over a range of reasonable half-lives.

One of the general recommendations of Cooper et al. (2016) was to pay attention to parameter estimates and their uncertainty to avoid error in analyses. Yet they misinterpreted their own results by neglecting units, scale, meaning, and estimation uncertainty of α. The main lesson to be drawn from their analyses is not that there are problems with fitting Ornstein–Uhlenbeck models, but rather that model-selection and interpretation should not be based solely on significance testing (see also Amrhein et al. 2019; Wasserstein et al. 2019).

## Misconceptions about measurement error

Using a similar setup as above, Cooper et al. (2016) further simulated the effects of error in the species values on the rejection rates. In comparative analyses, “measurement error” usually refers to estimation error in the species means. The variance of these errors can be quantified by the (squared) standard error of the estimate (Hansen & Bartoszek 2012) and includes both natural variance among individuals and variance in the measurements of these individuals. For brevity and consistency with Cooper et al. (2016), we will use “measurement error” as an umbrella term that can refer to any source of error or error variance in species means. Cooper et al. (2016) claimed that with as little as 1% of “measurement error,” the Ornstein–Uhlenbeck model was often inappropriately chosen over the Brownian-motion model and this error frequency increased with the size of the tree. Based on these results they claimed that “we cannot conclude anything about the evolutionary process from a single optimum [Ornstein–Uhlenbeck] model unless error is adequately accounted for” (p. 69).

Why is the Ornstein–Uhlenbeck model preferred with increasing error and larger trees? The reason is conceptually simple. Measurement error increases among-species variance relative to phylogenetic covariance (e.g., Lynch 1991; Felsenstein 2008; Hansen and Bartoszek 2012), and this will reduce the phylogenetic signal. When fitting an Ornstein–Uhlenbeck model, the reduction in phylogenetic signal will manifest as a shorter phylogenetic half-life. If the only choice is between pure Ornstein–Uhlenbeck and Brownian-motion models, lowering the phylogenetic half-life will increase support for the Ornstein–Uhlenbeck model. Moreover, with more tips, there is more power to reject Brownian motion. This is not a flaw of the Ornstein–Uhlenbeck model, because in models with a single optimum, the half-life describes the correlation structure in the data. The same effect will appear with other measures of phylogenetic signal, such as phylogenetic heritability (Lynch 1991; Housworth et al. 2004).

The effect of measurement error on parameter estimation in comparative methods is important and has been extensively discussed (e.g., Martins and Hansen 1997; Ives et al. 2007; Felsenstein 2008; Hansen and Bartoszek 2012; Garamszegi 2014; Silvestro et al. 2015; Grabowski et al. 2016). This is not a problem particular to Ornstein–Uhlenbeck models, however; it applies to any model-based comparative inference. Furthermore, many modern applications of phylogenetic comparative methods, including those based on the Ornstein–Uhlenbeck model, allow for modeling of measurement error (e.g., Hansen et al. 2008; Bartoszek et al. 2012; Beaulieu et al. 2012; Ho and Ané 2014b; Uyeda and Harmon 2014). Indeed, many of these applications were available at the time of Cooper et al.’s (2016) analyses, but they did not take the opportunity to investigate the performance of approaches that accommodate measurement error. To reinforce our point, we have included a relatively simple example in the supplementary material where we simulate traits under Brownian motion and inferred the half-life under a range of conditions—varying tree size, measurement error, and whether the model accounted for measurement error. Our results show that not considering observation error can lead to incorrect inferences, but more importantly that accounting for error variance in the model alleviates the problem (see Supplementary Text S1).

## Conclusions

Investigating parameter identifiability, goodness of fit, and other properties of phylogenetic comparative models are all valuable efforts (Pennell et al. 2015; Uyeda et al. 2018). Yet, we emphasize that Cooper et al.’s (2016) note has little relevance to the use of Ornstein–Uhlenbeck models, due to three fundamental misinterpretations. First, by only considering single-optimum models, they provided no evidence of problems relevant to the macroevolutionary analysis of adaptation, which is *the* key application of the Ornstein–Uhlenbeck model in comparative analyses. Second, by neglecting parameter interpretation and uncertainty, they provided no evidence for problems in using the single-optimum Ornstein–Uhlenbeck model to estimate phylogenetic signal. Third, by not considering measures of estimation uncertainty, methods to incorporate error, and previous studies of the subject, they provided no new insights into the effects of measurement error in comparative analyses.

We reiterate a point that may be lost on researchers who are not specialists in phylogenetic comparative methods: the Ornstein–Uhlenbeck process is a continuum of models, moving from instantaneous adaptation to Brownian motion. Thus, the debate about the Ornstein–Uhlenbeck process versus Brownian motion is largely fictitious. If adaptation has been slow, data will conform to a pattern of Brownian motion, although possibly including trends. More often the converse may hold. Fast adaptation will erase phylogenetic signal in the model residuals, and the Ornstein–Uhlenbeck model will converge on standard non-phylogenetic regression or ANOVA. Hence, simply assuming a particular phylogenetic effect a priori—equivalent to drawing a qualitative conclusion when the data allow a quantitative estimate—robs the data of their ability to tell a more nuanced, interesting, and biologically realistic story (e.g. see Bartoszek et al. (2022) for a timely example of how the multivariate Ornstein–Uhlenbeck process provides new insights to coadaptation and evolutionary trade-offs). Another point worth repeating is the importance of fitting models appropriate to the hypothesis under investigation. Brownian motion may be more tractable, but tractability matters little if a model is inappropriate for answering our research question. What is the point of estimating parameters if the parameters cannot tell us what we want to know?

## Supplementary Material

Data available from the Dryad Digital Repository: http://dx.doi.org/10.5061/dryad.jwstqjqc1.

## References

[CIT0001] Amrhein V. , Greenland S., McShane B. 2019. Retire statistical significance. Nature 567:305–307. 30894741 10.1038/d41586-019-00857-9

[CIT0002] Bartoszek K. , Fuentes-Gonzales J.A., Mitov V., Pienaar J., Piwczynski M., Puchalka R., Spalik K., Voje K.L. 2022. Model selection performance in phylogenetic comparative methods under multivariate Ornstein-Uhlenbeck models of trait evolution. Syst. Biol.: syac079. doi:10.1093/sysbio/syac079. PMC11302515 36575879

[CIT0003] Bartoszek K. , Pienaar J., Mostad P., Andersson S., Hansen T.F. 2012. A phylogenetic comparative method for studying multivariate adaptation. J. Theor. Biol. 314:204–215. 22940235 10.1016/j.jtbi.2012.08.005

[CIT0004] Bastide P. , Ané C., Robin S., Mariadassou M. 2018. Inference of adaptive shifts for multivariate correlated traits. Syst. Biol. 67:662–680. 29385556 10.1093/sysbio/syy005

[CIT0005] Beaulieu J.M. , Jhwueng D.-C., Boettiger C., O’Meara B.C. 2012. Modeling stabilizing selection: expanding the Ornstein-Uhlenbeck model of adaptive evolution. Evolution 66:2369–2383. 22834738 10.1111/j.1558-5646.2012.01619.x

[CIT0006] Boettiger C. , Coop G., Ralph P. 2012. Is your phylogeny informative? Measuring the power of comparative methods. Evolution 66:2240–2251. 22759299 10.1111/j.1558-5646.2011.01574.x PMC3448289

[CIT0007] Butler M.A. , King A.A. 2004. Phylogenetic comparative analysis: a modeling approach for adaptive evolution. Am. Nat. 164:683–695. 29641928 10.1086/426002

[CIT0008] Clavel J. , Escarguel G., Merceron G. 2015. mvMORPH: an R package for fitting multivariate evolutionary models to morphometric data. Meth. Ecol. Evol. 6:1311–1319.

[CIT0009] Cooper N. , Thomas G.H., Venditti C., Meade A., Freckleton R.P. 2016. A cautionary note on the use of Ornstein Uhlenbeck models in macroevolutionary studies. Biol. J. Linn. Soc. Lond. 118:64–77. 27478249 10.1111/bij.12701 PMC4949538

[CIT0010] Cornuault J. 2022. Bayesian analyses of comparative data with the Ornstein–Uhlenbeck model: potential pitfalls. Syst. Biol. 71:1524–1540. 35583306 10.1093/sysbio/syac036 PMC9558839

[CIT0011] Cressler C.E. , Butler M.A., King A.A. 2015. Detecting adaptive evolution in phylogenetic comparative analysis using the Ornstein–Uhlenbeck model. Syst. Biol. 64:953–968. 26115662 10.1093/sysbio/syv043

[CIT0012] Dominicus A. , Skrondal A., Gjessing H.K., Pedersen N.L., Palmgren J. 2006. Likelihood ratio tests in behavioral genetics: problems and solutions. Behav. Genet. 36:331–340. 16474914 10.1007/s10519-005-9034-7

[CIT0013] Felsenstein J. 2008. Comparative methods with sampling error and within‐species variation: contrasts revisited and revised. Am. Nat. 171:713–725. 18419518 10.1086/587525

[CIT0014] Garamszegi L.Z. 2014. Uncertainties due to within-species variation in comparative studies: measurement errors and statistical weights. In: Garamszegi L.Z., editor. Modern phylogenetic comparative methods and their application in evolutionary biology. Berlin, Heidelberg: Springer. p. 157–199.

[CIT0015] Grabowski M. , Kopperud B.T., Tsuboi M., Hansen T.F. 2023. Both diet and sociality affect primate brain-size evolution. Syst. Biol. 10.1093/sysbio/syac075 PMC10275546 36454664

[CIT0016] Grabowski M. , Voje K.L., Hansen T.F. 2016. Evolutionary modeling and correcting for observation error support a 3/5 brain-body allometry for primates. J. Hum. Evol. 94:106–116. 27178462 10.1016/j.jhevol.2016.03.001

[CIT0017] Hansen T.F. 1997. Stabilizing selection and the comparative analysis of adaptation. Evolution 51:1341–1351. 28568616 10.1111/j.1558-5646.1997.tb01457.x

[CIT0018] Hansen T.F. 2014. Use and misuse of comparative methods in the study of adaptation. In: Garamszegi L.Z., editor. Modern phylogenetic comparative methods and their application in evolutionary biology. Berlin, Heidelberg: Springer. p. 351–379.

[CIT0019] Hansen T.F. , Bartoszek K. 2012. Interpreting the evolutionary regression: the interplay between observational and biological errors in phylogenetic comparative studies. Syst. Biol. 61:413–425. 22213708 10.1093/sysbio/syr122

[CIT0020] Hansen, T.F., Orzack, S.H. 2005. Assessing current adaptation and phylogenetic inertia as explanations of trait evolution: the need for controlled comparisons. Evolution 59:2063–2072. 16405152

[CIT0021] Hansen T.F. , Pienaar J., Orzack S.H. 2008. A comparative method for studying adaptation to a randomly evolving environment. Evolution 62:1965–1977. 18452574 10.1111/j.1558-5646.2008.00412.x

[CIT0022] Hipp A.L. , Escudero M. 2010. MATICCE: mapping transitions in continuous character evolution. Bioinformatics 26:132–133. 19880368 10.1093/bioinformatics/btp625

[CIT0023] Ho L.S.T. , Ané C. 2013. Asymptotic theory with hierarchical autocorrelation: Ornstein–Uhlenbeck tree models. Ann. Stat. 41:957–981.

[CIT0024] Ho L.S.T. , Ané C. 2014a. A linear-time algorithm for Gaussian and non-Gaussian trait evolution models. Syst. Biol. 63:397–408. 24500037 10.1093/sysbio/syu005

[CIT0025] Ho L.S.T. , Ané C. 2014b. Intrinsic inference difficulties for trait evolution with Ornstein-Uhlenbeck models. Meth. Ecol. Evol. 5:1133–1146.

[CIT0026] Höhna S. , May M.R., Moore B.R. 2016. TESS: an R package for efficiently simulating phylogenetic trees and performing Bayesian inference of lineage diversification rates. Bioinformatics 32:789–791. 26543171 10.1093/bioinformatics/btv651

[CIT0027] Housworth E.A. , Martins E.P., Lynch M. 2004. The phylogenetic mixed model. Am. Nat. 163:84–96. 14767838 10.1086/380570

[CIT0028] Hunt G. , Bell M.A., Travis M.P. 2008. Evolution toward a new adaptive optimum: phenotypic evolution in a fossil stickleback lineage. Evolution 62:700–710. 18081713 10.1111/j.1558-5646.2007.00310.x

[CIT0029] Ingram T. , Mahler D.L. 2013. SURFACE: detecting convergent evolution from comparative data by fitting Ornstein-Uhlenbeck models with stepwise Akaike Information Criterion. Meth. Ecol. Evol. 4:416–425.

[CIT0030] Ives A.R. , Midford P.E., Garland T. 2007. Within-species variation and measurement error in phylogenetic comparative methods. Syst. Biol. 56:252–270. 17464881 10.1080/10635150701313830

[CIT0031] Khabbazian M. , Kriebel R., Rohe K., Ané C. 2016. Fast and accurate detection of evolutionary shifts in Ornstein-Uhlenbeck models. Meth. Ecol. Evol. 7:811–824.

[CIT0032] Kopperud B.T. , Pienaar J., Voje K.L., Orzack S.H., Hansen T.F. 2020. slouch: Stochastic Linear Ornstein-Uhlenbeck Comparative Hypotheses. R package version 2.1.4.

[CIT0033] Labra A. , Pienaar J., Hansen T.F. 2009. Evolution of thermal physiology in *Liolaemus* lizards: adaptation, phylogenetic inertia, and niche tracking. Am. Nat. 174:204–220. 19538089 10.1086/600088

[CIT0034] Lo Cascio Sætre C. , Coleiro C., Austad M., Gauci M., Sætre G.-P., Voje K.L., Eroukhmanoff F. 2017. Rapid adaptive phenotypic change following colonization of a newly restored habitat. Nat. Commun. 8:14159. 28106055 10.1038/ncomms14159 PMC5263874

[CIT0035] Lynch M. 1991. Methods for the analysis of comparative data in evolutionary biology. Evolution 45:1065–1080. 28564168 10.1111/j.1558-5646.1991.tb04375.x

[CIT0036] Magallón S. , Sanderson M.J. 2001. Absolute diversification rates in angiosperm clades. Evolution 55:1762–1780. 11681732 10.1111/j.0014-3820.2001.tb00826.x

[CIT0037] Mahler D.L. , Ingram T. 2014. Phylogenetic comparative methods for studying clade-wide convergence. In: L.Z. Garamszegi, editor. Modern phylogenetic comparative methods and their application in evolutionary biology: concepts and practice. Berlin, Heidelberg: Springer. p. 425–450

[CIT0038] Martins E.P. 2000. Adaptation and the comparative method. Trends Ecol. Evol. 15:296–299. 10856957 10.1016/s0169-5347(00)01880-2

[CIT0039] Martins E.P. , Hansen T.F. 1997. Phylogenies and the comparative method: a general approach to incorporating phylogenetic information into the analysis of interspecific data. Am. Nat. 149:646–667.

[CIT0040] Mitov V. , Bartoszek K., Asimomitis G., Stadler T. 2020. Fast likelihood calculation for multivariate Gaussian phylogenetic models with shifts. Theor. Pop. Biol. 131:66–78. 31805292 10.1016/j.tpb.2019.11.005

[CIT0041] Moen D.S. , Cabrera-Guzmán E., Caviedes-Solis I.W., González-Bernal E., Hanna A.R. 2022. Phylogenetic analysis of adaptation in comparative physiology and biomechanics: overview and a case study of thermal physiology in treefrogs. J. Exp. Biol. 225:jeb243292. 35119071 10.1242/jeb.243292

[CIT0042] O’Meara B. , Beaulieu J. 2014. Modelling stabilizing selection: the attraction of Ornstein-Uhlenbeck models. In: L.Z. Garamszegi, editor. Modern phylogenetic comparative methods and their application in evolutionary biology: concepts and practice. Berlin, Heidelberg: Springer. p. 381–394.

[CIT0043] Ota R. , Waddell P.J., Hasegawa M., Shimodaira H., Kishino H. 2000. Appropriate likelihood ratio tests and marginal distributions for evolutionary tree models with constraints on parameters. Mol. Biol. Evol. 17:798–803. 10779540 10.1093/oxfordjournals.molbev.a026358

[CIT0044] Pennell M.W. , FitzJohn R.G., Cornwell W.K., Harmon L.J. 2015. Model adequacy and the macroevolution of angiosperm functional traits. Am. Nat. 186:E33–E50. 26655160 10.1086/682022

[CIT0045] Revell L.J. 2010. Phylogenetic signal and linear regression on species data. Meth. Ecol. Evol. 1:319–329.

[CIT0046] Ross C.T. , Strimling P., Ericksen K.P., Lindenfors P., Mulder M.B. 2016. The origins and maintenance of female genital modification across Africa: Bayesian phylogenetic modeling of cultural evolution under the influence of selection. Hum. Nat. 27:173–200. 26846688 10.1007/s12110-015-9244-5

[CIT0047] Self S.G. , Liang K.-L. 1987. Asymptotic properties of maximum likelihood estimators and likelihood ratio tests under nonstandard conditions. J. Am. Stat. Assoc. 82:605–610.

[CIT0048] Silvestro D. , Kostikova A., Litsios G., Pearman P.B., Salamin N. 2015. Measurement errors should always be incorporated in phylogenetic comparative analysis. Meth. Ecol. Evol. 6:340–346.

[CIT0049] Thomas G.H. , Freckleton R.P. 2012. MOTMOT: models of trait macroevolution on trees. Meth. Ecol. Evol. 3:145–151.

[CIT0050] Uyeda J.C. , Harmon L.J. 2014. A novel bayesian method for inferring and interpreting the dynamics of adaptive landscapes from phylogenetic comparative data. Syst. Biol. 63:902–918. 25077513 10.1093/sysbio/syu057

[CIT0051] Uyeda J.C. , Pennell M.W., Miller E.T., Maia R., McClain C.R. 2017. The evolution of energetic scaling across the vertebrate tree of life. Am. Nat. 190:185–199. 28731792 10.1086/692326

[CIT0052] Uyeda J.C. , Zenil-Ferguson R., Pennell M.W. 2018. Rethinking phylogenetic comparative methods. Syst. Biol. 106:13410–13419. 10.1093/sysbio/syy031 29701838

[CIT0053] Voje K.L. 2020. Testing eco-evolutionary predictions using fossil data: phyletic evolution following ecological opportunity. Evolution 74:188–200. 31461158 10.1111/evo.13838

[CIT0054] Wasserstein R.L. , Schirm A.L., Lazar N.A. 2019. Moving to a world beyond “ p < 0.05.”. Am. Stat. 73:1–19.

